# The Chemical Composition and Antibacterial and Antioxidant Activities of Five Citrus Essential Oils

**DOI:** 10.3390/molecules27207044

**Published:** 2022-10-19

**Authors:** Yan Li, Shutian Liu, Chen Zhao, Zhuo Zhang, Dechao Nie, Weixuan Tang, Yanling Li

**Affiliations:** Animal Science and Technology College, Beijing University of Agriculture, No. 7 Beinong Road, Changping, Beijing 102206, China

**Keywords:** citrus essential oil, antibacterial, antioxidant

## Abstract

Increasing concerns over the use of antimicrobial growth promoters in animal production has prompted the need to explore the use of natural alternatives such as phytogenic compounds and probiotics. Citrus EOs have the potential to be used as an alternative to antibiotics in animals. The purpose of this research was to study the antibacterial and antioxidant activities of five citrus EOs, grapefruit essential oil (GEO), sweet orange EO (SEO), bergamot EO (BEO), lemon EO (LEO) and their active component d-limonene EO (DLEO). The chemical composition of EOs was analyzed by gas chromatography–mass spectrometry (GC-MS). The antibacterial activities of the EOs on three bacteria (*Escherichia coli*, *Salmonella* and *Lactobacillus acidophilus*) were tested by measuring the minimum inhibitory concentration (MIC), minimal bactericidal concentration (MBC) and inhibition zone diameter (IZD). The antioxidant activities of EOs were evaluated by measuring the free radical scavenging activities of DPPH and ABTS. We found that the active components of the five citrus EOs were mainly terpenes, and the content of d-limonene was the highest. The antibacterial test showed that citrus EOs had selective antibacterial activity, and the LEO had the best selective antibacterial activity. Similarly, the LEO had the best scavenging ability for DPPH radicals, and DLEO had the best scavenging ability for ABTS. Although the main compound of the five citrus EOs was d-limonene, the selective antibacterial and antioxidant activity of them might not be primarily attributed to the d-limonene, but some other compounds’ combined action.

## 1. Introduction

During the last decades, plant-derived essential oils (EOs) have attracted extensive attention as potential substitutes for antibiotic growth promoters (AGPs) in animal production because of their antibacterial, antioxidant and anti-inflammatory activities [1,2]. Supplementation of EOs has been reported for improving the intestinal absorption of essential nutrients, stimulating blood circulation, exerting antioxidant performance and reducing the number of pathogenic bacteria and the activities of the intestinal-related lymphatic system [2,3,4]. Most EOs have broad-spectrum antibacterial activity, which can effectively inhibit harmful bacteria such as *E. coli*, but also affect beneficial bacteria such as *Lactobacillus* [5]. Therefore, selecting drugs with selective antibacterial activity can inhibit pathogenic bacteria without harming beneficial bacteria or having less impact on beneficial bacteria, which is particularly important for improving the structure of intestinal flora and protecting an animal’s body. In addition, throughout the model of livestock production, animals are often exposed to stressful conditions resulting from nutrition (e.g., high grain), environment and management (weaning, transportation, feedlot entry). Stressful events have been implicated in promoting oxidative stress through excessive reactive oxygen species (ROS) production or decreased antioxidant defenses [6]. Oxidative stress can result in DNA hydroxylation, protein denaturation, lipid peroxidation and further lead to apoptosis [7]. The oxidative stress could also cause intestinal and metabolic diseases in animals [8], resulting from oxidative damage and immune deficiency in the animal organism. Some synthetic antioxidants, such as butyl hydroxytoluene (BHT) and butyl hydroxyanisole (BHA), are commonly used in animal production, but they might have carcinogenic potential [8]. Supplementing EOs in animal feed may be a nutritional strategy to prevent damage to the animal body caused by oxidative stress, because of their antioxidant properties [9]. However, until now most of studies using EOs have been focused on its antimicrobial activity in rumen, and its antioxidant activity was often overlooked.

The genus citrus is a family of Rutaceae, and mainly includes orange, lemon, grapefruit, limes and many other plants. Citrus EOs are primarily extracted from the peel and are the most produced plant EOs in the world, accounting for about 1/3 of the total EOs [10]. The bioactive components of citrus EOs are primarily monoterpenes such as d-limonene and less so sesquiterpenes, their oxygenated derivatives [11]. These volatile compounds are believed to play critical roles due to their biological activities such as antibacterial and antioxidant activity [11,12]. Citrus EOs can prevent animal diseases caused by microbial pathogens and oxidative damage to the body because of their antibacterial, antioxidant and anti-inflammatory activities, as well as other biological activities [13]. They might have the potential to replace AGPs as natural antioxidants. In addition, studies have found that some citrus EOs have selective antibacterial activity against pathogenic bacteria and beneficial bacteria, such as Brazilian orange terpenes [14]. However, there are few studies on the selective antibacterial activity of the EOs derived from grapefruit, sweet orange, bergamot, or lemon. Therefore, the purpose of this study was to determine the chemical components and the selective antibacterial and antioxidant abilities of grapefruit EO (GEO), sweet orange EO (SEO), bergamot EO (BEO), lemon EO (LEO) and the d-limonene EO (DLEO).

## 2. Results

### 2.1. Chemical Composition of Essential Oils

The chemical composition of the citrus EOs is shown in Table 1. There were 20, 25, 31, 30, and 20 different active components that were detected in GEO, SEO, BEO, LEO, and DLEO, respectively. The most abundant components in GEO, SEO, BEO, LEO, and DLEO were monoterpene olefins, with 90.27%, 89.18%, 48.3%, 86.58% and 96.19%, respectively. D-limonene was the main component, with the contents of 81.86%, 78.30%, 34.23%, 47.34% and 80.73% in total for GEO, SEO, BEO, LEO and DLEO, respectively. Linalyl acetate (27.69%) in BEO was also high, second only to limonene, and these two components alone accounted for 61.92% of the total active components determined. In addition, a small number of common compounds were also detected, such as cis-limonene oxide, carvone, trans-limonene oxide, and p-menthol-1(7),8-diene, which were small molecules but may also confer biological activity to citrus EOs.

### 2.2. Antibacterial Activity

#### 2.2.1. Minimum Inhibitory Concentration (MIC) and Minimum Bactericidal Concentration (MBC) of Citrus EOs

The MIC and MBC of the EOs against *E. coli*, *Salmonella* and *L. acidophilus* are shown in Table 2. Although no statistical analysis was able to be conducted, the obvious differences in MIC among EOs were obvious with *E. coli* and *Salmonella*. For *E. coli*, the MIC was roughly the lowest with GEO and LEO, intermediate with DLEO and highest with SEO and BEO, while for *Salmonella*, the MIC was lowest with DLEO and SEO, intermediate with GEO and BEO and highest with LEO. However, there were no differences in MIC among EOs for *L. acidophilus*, except DLEO which had a lower MIC than other EOs. Regarding the MBC, as expected, the MBC was much higher than MIC, but the similar trends among EOs were followed between MIC and MBC for *E. coli* and *L. acidophilus*, whereas the MBC was lowest with DLEO and LEO, intermediate with SEO and BEO and highest with GEO.

#### 2.2.2. Inhibition Zone Diameter (IZD) of EOs

The IZDs of EOs are shown in Table 3. The tested strains were sensitive to all citrus EOs (IZD > 7). The IZD of LEO against *E. coli* was higher than that of the GEO (*P* = 0.01) and SEO (*P* = 0.03), but without difference (*P* > 0.05) with that of BEO and DLEO. There was no difference (*P* > 0.05) in the IZD of all EOs against *Salmonella*. For *L. acidophilus*, the IZD of LEO was lower (*P* < 0.05) than that of the other four EOs, whereas the IZDs of GEO and SEO were higher (*P* < 0.01) than that of BEO, and the IZD of GEO was greater (*P* < 0.01) than that of DLEO.

### 2.3. Antioxidant Activity

#### 2.3.1. Scavenging Activity of Citrus EOs on DPPH Free Radical

**Figure 1:**
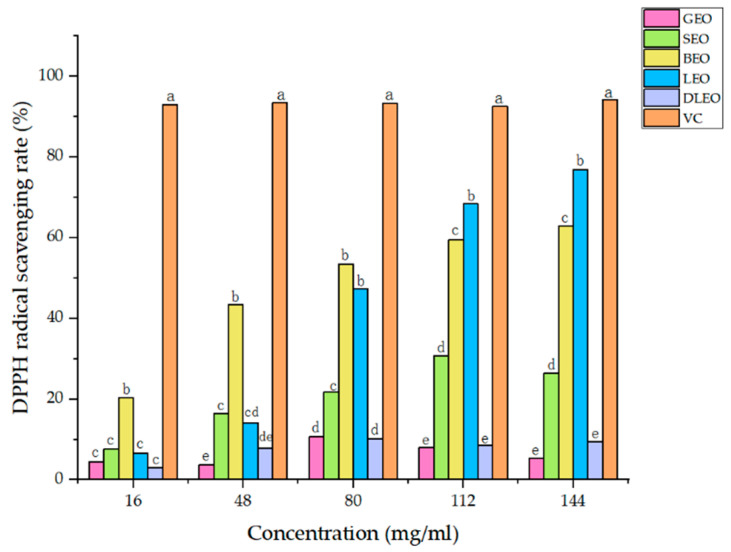
Effect of EO type and concentration on DPPH radical scavenging rate. Different EOs (E) and concentrations (C) had significant effects on DPPH radical scavenging rate (*P*_E_ < 0.01, *P*_C_ < 0.01), and their interaction (E × C) was significant (*P*_E_ × *P*_C_ < 0.01). Different letters show differences (*P* < 0.05) among EOs. GEO: grapefruit essential oil, SEO: sweet orange essential oil, BEO: bergamot essential oil, LEO: lemon essential oil, DLEO: d-limonene essential oil, VC: Vitamin C.

Figure 1 shows the effect of the EO source and the EO dosage on the scavenging rates of DPPH radicals. The interactions of EO type and concentration were significant (*P* < 0.01) on the DPPH radical scavenging rate. The DPPH radical scavenging rate quadratically increased (*P_Q_* < 0.01) with increasing the concentration of SEO, BEO and DLEO, and linearly increased (*P_L_* < 0.01) with increasing the concentration of LEO, with no difference (*P* > 0.05) in the DPPH radical scavenging rate with increasing the concentrations of GEO. At the concentration of 16 mg/mL, the DPPH radical scavenging rate was higher (*P* < 0.05) with BEO than the other EOs. At the concentration of 48 mg/mL, the DPPH radical scavenging rates were higher (*P* < 0.05) with SEO and LEO than with DLEO and GEO, but there was no difference (*P* > 0.05) in the DPPH free radical scavenging rate between SEO and LEO. At the concentration of 80 mg/mL, the DPPH radical scavenging rates were highest (*P* < 0.05) with BEO and LEO, intermediate with SEO and lowest with GEO and DLEO. At the concentrations of 112 and 144 mg/mL, LEO had the highest (*P* < 0.05) DPPH radical scavenging rate, followed by BEO, and lowest with GEO and DLEO. The DPPH radical scavenging rate of VC did not vary with its increasing concentration.

#### 2.3.2. Scavenging Activity of Citrus EOs on ABTS Free Radical

**Figure 2:**
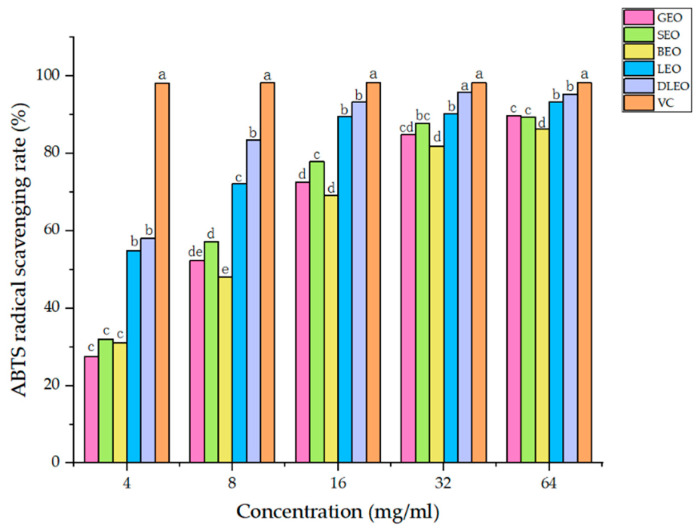
Effect of EO type and concentration on ABTS radical scavenging rate. Type of EO (E) and its concentration (C) had significant effects on ABTS radical scavenging rate (*P*_E_ < 0.01, *P*_C_ < 0.01), and their interaction (E × C) was significant (*P*_E_ × *P*_C_ < 0.01). Different letters show differences (*P* < 0.05) among EOs. GEO: grapefruit essential oil, SEO: sweet orange essential oil, BEO: bergamot essential oil, LEO: lemon essential oil, DLEO: d-limonene essential oil, VC: Vitamin C.

Figure 2 shows the results of EO type and concentration on the scavenging rate of ABTS radicals. The interaction of EO type with concentration was noticed (*P* < 0.01) on the ABTS radical scavenging rate. The ABTS radical scavenging rate of SEO, BEO, GEO, LEO and DLEO quadratically increased (*P_Q_* < 0.01) with increasing EO concentration, with no difference (*P* > 0.05) in the ABTS radical scavenging rate with the increasing concentrations of VC. In comparing among EOs, the ABTS radical scavenging rate differed (*P* < 0.05) at a concentration of 4 mg/mL (LEO, DLEO > GEO, SEO, BEO), at the concentration of 8 (DLEO > LEO > GEO, SEO, BEO; GEO > BEO), at the concentration of 16 (DLEO > LEO > SEO > GEO, BEO), at the concentration of 32 (DLEO > 4 EOs; LEO > GEO, BEO; SEO > BEO) and at the concentration of 64 (DLEO, LEO > GEO, SEO > BEO). The VC had consistently greater scavenging rate of ABTS compared with all EOs.

## 3. Discussion

The bioactivity of citrus EOs has been widely studied, but mainly in the food, cosmetics and pharmaceutical industries. Recently, in animal production, it has become a trend to find a natural antibacterial agent as a substitute for antibiotic growth promoters and antioxidants. The selectivity of individual components of EOs and EOs to pathogenic and beneficial bacteria have been reported [15]. EOs or most single components of EOs showed high efficacy against *Salmonella typhimurium*, *E. coli O157: H7* and *E. coli K88*, but had little inhibitory effect on *Lactobacillus* and *Bifidobacterium*. Bhandari et al. [16] found that citrus EOs showed selective antibacterial activity in vitro. In agreement with those studies, the present study showed that the citrus EOs had selective antibacterial activity and inhibitory activity against pathogenic *E. coli* and *Salmonella*, but their activity varied with type of EO and the concentration added.

The active components of EO affects the biological activity [17]. D-limonene has been proven to be the main compound of citrus EOs, accounting for the largest proportion of components [18]. The results of Dosoky et al. [19] showed that sweet orange EO, bitter orange EO, citrus EO and grapefruit EO were rich in monoterpenoids, and the main component was d-limonene. In this study, d-limonene was the main active compound of GEO, SEO, LEO and DLEO, while d-limonene and linalyl acetate were the main compounds of BEO. Other researchers found that BEO was mainly composed of linalool acetate and linalool [20,21]. Djenane et al. [20] showed that monoterpenes were the main compounds (97%) of citrus EOs, while the other compounds, such as alcohols, aldehydes and esters, were only ranging from 1.8 to 2.2%. However, in our study, the monoterpene content of the five citrus EOs was less than 97%, and only 48.3% was contained in BEO. In fact, the composition of EOs could be affected by fruit maturity, plant growth stage, storage conditions, extraction methods and so on [22]. Vekiari et al. [23] analyzed the chemical composition of LEO and found high contents of β-pinene (21.2%), γ-pinene (17.4%) and α-pinene (9.8%), in addition to d-limonene. In contrast, the β-pinene (13.74%), γ-pinene (10.55%) and β-laurene (4.20%) contents of LEO were lower in our study. The differences in the active components of EOs between the present and other studies might be due to variation in the genetics, age and growing environment of plants [24].

The ability of citrus EOs to inhibit pathogens has been reported in many studies [13]. Yi et al. [25] reported the inhibitory activity of citrus EO (Nanfeng mandarin) on *E. coli*, which was moderately sensitive to citrus EO (IZD at 11 to 15 mm). In our study, *E. coli* was also moderately sensitive to five citrus EOs and the IZD was between 10–5 mm. Deng et al. [26] found that the sensitivity of different bacteria to GEO was ranked as follows: *B. subtilis* > *E. coli* > *S. aureus* > *S. typhimurium* > *P. aeruginosa*. These results are in agreement with the present study that the antibacterial effect of GEO against *E. coli* was better than that of *Salmonella*. It suggests that the antimicrobial activity of citrus EOs was strain-dependent [27]. Viuda-martos et al. [28] found that orange, lemon, mandarin and grapefruit EOs had lower inhibitory activity against *Lactobacillus curvatus* and *L. sakei*, which was confirmed by the present study, with the lowest inhibitory effect against these two bacteria at the highest concentration. Ambrosio et al. [29] reported that orange oil phase essence and citrus terpens had the highest inhibitory activity against pathogenic bacteria (*E. coli*, *S. aureus*, *E. faecalis*, *Listeria monocytogenes*) and the lowest inhibitory activity against beneficial bacteria (*Lactobacillus plantarum* and *Lactobacillus rhamnosus*). A similar result was observed in our study, where LEO showed the lowest inhibitory activity against *L. acidophilus* (8.67 ± 1.53 mm), and it was inhibited only at the high concentrations tested. It showed that citrus EOs had selective antibacterial activity against the most pathogenic and beneficial bacteria. Although the general structure and biosynthetic pathways of Gram-positive bacteria are conserved, such as *Lactobacillus*, some Gram-positive bacteria (*L. acidoplhilus*) might have a lower susceptibility to EOs, because the cell wall of Gram-positive lactic acid bacteria has unique properties that could confer resistance to certain antimicrobial agents [30]. For instance, the inherent resistance of some *Lactobacilli* to antibiotics (e.g., to vancomycin) was related to the fact that D-lactate or D-serine replace D-alanine as the last amino acid in the peptidoglycan layer peptide chain of their cell wall [30,31], which would prevent the antimicrobial agent from binding to the peptide chain and lead to the inhibition of these bacteria [32]. The tolerance of *L. acidoplhilus* to d-limonene may be similar, but the exact mechanism needs to be further investigated in depth.

The EOs of tangerine, grapefruit, lemon and cinnamon have different degrees of antibacterial activity against all pathogenic microorganisms tested, because of their different chemical components [33]. Antibacterial activity also depends on the volatility, stability and hydrophobicity of compounds of EO, such as limonene, which was highly volatile, easy to oxidize and has low solubility in water [34]. Therefore, the high content of d-limonene might not produce high antibacterial activity. Bourgou et al. [35] found that the main active component of bitter orange EO, LEO, malt orange EO and citrus EO was the monoterpene d-limonene, and the antibacterial activity of four citrus EOs appeared not to be associated with the content of d-limonene. In this experiment, although the d-limonene content of DLEO was higher than that of LEO, the antibacterial activity of DLEO was lower than that of LEO. It has also been reported that *Citrus limon var. pompia* leaf EO had inhibitory activity against pathogenic bacteria, whereas d-limonene had no inhibitory activity against pathogenic bacteria [36]. It suggested that the antibacterial activity of citrus EOs might not be attributed solely by d-limonene, but it may be due to the complex interaction among components. Other oxygenated monoterpenes, such as α-pinoresinol, cis-geraniol, β-citral, nerolidol and α-citral, might also be associated with the antibacterial activity of citrus EOs, as they are present at high contents in citrus EOs with high antibacterial activity [37]. In our study, the most selective antibacterial activity of citrus EO also detected small amounts of oxygenated monoterpenes (e.g., cis-citral, α-pine deprenyl, trans-citral), and these compounds might play an important role in the selective antibacterial activity of citrus EOs. In addition, the antibacterial activity of limonene has been shown to be variable and dependent on the stereoisomeric form present in EOs, with the (−) stereoisomer of limonene inhibiting *E. coli* at lower concentrations (8 mg/mL) compared to the (+) stereoisomer (11 mg/mL) [14].

It was reported that citrus EOs could also be used as a natural antioxidant [13]. Citrus EOs can reduce oxidative stress and prevent the oxidation of important biomolecules by scavenging free radicals [38]. Various in vitro antioxidant assay techniques have been used to assess antioxidant capacity, primarily through the ability to transfer hydrogen atoms and electron donors and the ability to chelate transition metals [39]. In our study, the antioxidant activity of five citrus EOs was assessed through the DPPH and ABTS free radical scavenging methods. At the same concentration, LEO and BEO had a good DPPH free radical scavenging rate, while SEO, DLEO and GEO had a poor scavenging rate, which indicated that the antioxidant activity of the EOs varied with the type of EOs. Lin et al. [40] found that four citrus EOs (Nanfeng mandarins, Xuanwu mandarins, Yangshuo kumquats and navel oranges) of southern China have different antioxidant activities. Similarly, Choi et al. [41] compared the DPPH free radical scavenging rate of 34 citrus EOs and found that 3 EOs showed a weak radical scavenging effect (17.7–19.1%), while another 31 EOs exhibited scavenging effects ranging from 21.6 to 64.0%. This difference could be explained by the different chemical composition of different varieties of citrus EOs. Al-Aamri et al. [42] reported that lime leaf EOs showed a dose-dependent increase in free radical scavenging activity, and the maximum response was reached at the highest concentration. A similar result was observed in our study, where five citrus EOs showed the DPPH and ABTS free radical scavenging rates in a dose-dependent manner and increased linearly or quadratically with increasing concentration. This indicates that the radical scavenging rate of citrus EOs varies with concentration. The changing position (crossing effect) of the free radical scavenging rate of the different EOs at different concentrations is worth noticing. Mau et al. [43] found that the scavenging effect of fraction 4 was higher than that of curcuma zedoaria EO at 1–10 mg/mL, but comparable to that of curcuma zedoaria EO at 15–20 mg/mL. However, the scavenging effects of BHA, ascorbic acid and α-tocopherol were much more effective at an extremely low concentration. This variation can be explained by the interaction between the type of EOs and the concentration of EOs. Frassinetti et al. [44] discovered that LEO showed the best antioxidant activity compared to bitter orange, sweet orange and mandarin EO. In our experiment, LEO also had a stronger DPPH free radical scavenging ability compared to the other four citrus EOs, and the ABTS free radicals scavenging rate of LEO and DLEO was comparable to that of the strong antioxidant VC. Therefore, LEO had the best antioxidant activity and had the potential to be used as a new green antioxidant in practical production.

The antioxidant activity of EOs is believed to be related to both their chemical composition and some main active compounds [45]. However, it is difficult to attribute the antioxidant effect of EOs only to one or several active compounds, because both secondary and primary compounds contribute greatly to the activity of EOs. Ambrosio et al. [46] found that secondary compounds play an important role in the biological activity of Brazilian orange terpenes, rather than only relying on the main compound limonene. Riahi et al. [47] reported that the major components of *Mentha rotundifolia* L. had low antioxidant capacity, while the minor components including pinocinol, β-pinene and caryophylene had high antioxidant capacity. Some terpenoids such as pineolene, α-terpinene and γ-terpinene have higher antioxidant properties than other terpenoids [48]. Teneva et al. [49] observed that citrus EOs had a stronger ability to scavenge DPPH free radicals than DLEO that contains primarily d-limonene, which suggests that the antioxidant activity of EO would result from the synergistic effect of the terpene mixture or the effect of non-volatile compounds. In our study, the DPPH clearance was overall higher with LEO and BEO than other EOs, which may be explained by their higher γ-terpinene profile of 10.55% and 4.51%, respectively. It suggests that the antioxidant activity of citrus EOs might be synergistic between d-limonene and γ-terpinene. We have also noticed that the LEO (10) and BEO (8) had more active components, which were >1% compared to SEO (6), GEO (6) and DLEO (5). It is speculated that a certain level of an individual component in the total concentration may be needed to effectively exhibit the synergistic activity. Therefore, the more the component is high in the profile of the EO, the greater activity of the EO would be expected. The components of LEO were higher than the other four EOs, which might explain the higher antioxidant activity of LEO. The proportion of γ-terpinene (4.51%) in BEO was higher than the other three EOs, which is consistent with the higher antioxidant activity. However, this speculation seems not to agree regarding the DLEO when the ABTS scavenging rates were measured, as it was the highest among the five citrus EOs in present study. The high ABTS scavenging rates of DLEO are likely not explained by the high d-limonene profile, because the SEO and GEO also had high d-limonene, but their ABTS scavenging rates were not high. These results suggest that the response of the active compound profile may be different depending on the type of antioxidant activity measured.

In summary, our results demonstrated that the selective antibacterial activity against pathogenic (*E. coli*, *Salmonella*) and beneficial bacteria (*L. acidophilus*) varied with type of citrus EOs. These properties could be used as an alternative to AGP in livestock production. These citrus EOs also had evident antioxidant activity for scavenging free radicals, whereas the antioxidant capacity varied with test methods used and type of EO and its dosage. By taking both antibacterial and antioxidant results into consideration, the LEO showed better antibacterial and antioxidant activities compared with the other EOs. It is speculated that a synergistic effect of multiple active components takes place instead of an individual component to exhibit the antibacterial and antioxidant activity.

## 4. Materials and Methods

### 4.1. Chemicals and Reagents

*E. coli* (ATCC25922), *Salmonella* and *L. acidophilus* strains were provided by the General Microbiology Center of China Microbial Strain Collection Management Committee (Beijing, China). Nutrient broth and all other chemicals were analytical grade.

### 4.2. Essential Oils and Active Component Analysis

The SEO, LEO, BEO, GEO and DLEO were evaluated in this study. The DLEO (0.8414 g/mL), SEO (0.8428 g/mL) and LEO (0.8468 g/mL) were extracted from the peel of the sweet orange and lemon and purchased from Nanjing Wensenbauer International Trade Co., Ltd. (Nanjing, China). The BEO (0.88 g/mL) and GEO (0.855 g/mL) were extracted from the peel of bergamot and grapefruit, and purchased from Poli Aromatic Pharmaceutical Technology Co., Ltd. (Shanghai, China). All five EOs were extracted from the citrus plant of the Rutaceae family.

The chemical constituents of the citrus EOs were analyzed using gas chromatography and mass spectrometry (GC-MS). The EO samples were diluted with n-hexane before analysis, and the active components were determined by GC-MS QP2010 ultra (Shimadzu, Kyoto, Japan). In brief, the diluted sample solution was separated by an Rxi-5Sil MS (30 m × 0.32 mm ID, 0.25 µm film thickness (Restek, Bellefonte, Newcastle, PA, USA)) capillary column. The column temperature was initially set at 50 °C for 5 min, then increased at a rate of 2 °C/min up to 320 °C and held for 5 min. The column temperature was initially set at 40 °C for 2 min, then increased at a rate of 6 °C/min up to 300 °C and held for 2 min. The injection volume of samples was 1 µL, with a split ratio of 10:1. High-purity helium was used as the carrier gas at a flow rate of 1 mL/min. The injector, transfer line and ion-source temperatures were 250, 280 and 220 °C, respectively. A solvent excision time of 3 min in the scan acquisition mode in the *m*/*z* 33–500 range was used. The data were collected by GC-MS solution 2.6 software (Shimadzu, Kyoto, Japan), and the accompanying solvent was blank in the process of sample detection. The data were screened and matched with NIST and other special standard spectrum libraries to identify individual component of EOs. Finally, the relative content was calculated by area normalization method.

### 4.3. Antibacterial Activity

#### 4.3.1. Determination of Minimum Inhibitory Concentration (MIC)

The MIC of citrus EOs was measured according to the method of Ambrosio [46] with some modifications using a 96-well microplate. The standard inoculum was prepared in sterile NaCl solution (0.9% *w*/*v*), and it was derived from the live colonies of the selected bacteria and contained in Mueller–Hinton (MH) agar (*E. coli* and *Salmonella*) or De Man Rogosa Sharpe (MRS) agar (*L. acidophilus*) plates with an optical density equal to 0.5 MacFarlane standard (values of 0.08–0.13 were read at 600 nm). Subsequently, the standard inoculum was diluted at 1:100 to obtain 10^6^ CFU/mL of inoculum. A sample solution of 160 mg/mL of EO was prepared using nutrient broth, and Tween 80 was used as an emulsifier. A two-fold series of dilutions of 100 µL of EO was first added to the 96-well plate, and the same volume of bacterial broth was added into each well. Broth culture was added to well A1 as blank control, and bacteria were added to well A2 as growth control, and three replicates were set for each treatment combination. Finally, *E. coli* and *Salmonella* were cultured at 37 °C for 24 h, and *L. acidophilus* at 30 °C for 36 h, and the absorbance was measured at 600 nm. The MIC value of the test well was determined when the difference between the test OD and blank OD value well was zero. The MIC was determined as the lowest concentration of EO that inhibited the growth of visible bacteria after incubation.

#### 4.3.2. Determination of Minimum Bactericidal Concentration (MBC)

Determination of MBC was performed from microplate wells with EO concentration where there was no visible bacterial growth. In brief, it was measured by collecting 100 µL aliquots (bacteria and EO mixture) from each well and inoculating them in MH or MRS agar. Then, the plates were incubated at 37 °C for 24 h for *E. coli* and *Salmonella*, and for 36 h for *L. acidophilus*. The MBC was defined as the lowest concentration of EO that causes all particular bacteria death.

#### 4.3.3. Determination of Inhibition Zone Diameter (IZD)

The IZD was determined using the disc diffusion method as described by Puškárová et al. [50]. Briefly, *E. coli* and *Salmonella* were inoculated on an MH agar plate, and *L. acidophilus* was inoculated on an MRS agar plate. Three sterile discs with diameters of 6 mm were placed in each plate and a disc (10 µL/disc) containing EOs (80 mg/mL) as treatment group. The MH agar plate and MRS agar plate were incubated in a thermostat incubator at 37 °C for 24 h. The diameter of inhibition zone (IZD) was measured with caliper after incubation, and the tests were carried out in triplicate. According to the drug sensitivity standard [29], the bacteria sensitivity to EO was classified according to the diameter of IZD: insensitive (IZD < 7 mm); low IZD between 7–9 mm; moderate IZD between 10–15 mm; high IZD between 15–20 mm; and extreme high IZD > 20 mm, as extremely sensitive.

### 4.4. Antioxidant Activity

#### 4.4.1. Experimental Design

The antioxidant activities of the five EOs at different concentrations were evaluated by measuring DPPH and ABTS free radical scavenging abilities. The experiment was a completed randomized design with 5 EOs (GEO, SEO, BEO, LEO and DLEO) × 5 concentrations of factorial arrangement of treatments. The concentration of each EOs in DPPH measurement was 16, 48, 80, 112 and 144 mg/mL and 4, 8, 16, 32 and 64 mg/mL in ABTS. Vitamin C with strong antioxidant activity was used as a positive control.

#### 4.4.2. DPPH Radical Scavenging Activity

The DPPH free radical scavenging activity was determined according to a previously described method [51]. 2,2-Diphenyl-1-picrylhydrazyl (DPPH) was dissolved in ethanol to obtain a concentration of 0.1 mmol/L, and then 100 μL of the DPPH solution was added to 100 μL of sample solution varying EO concentrations at 16, 48, 80, 112 and 144 mg/mL. The mixed solutions were incubated in the dark for 30 min and the OD values were read at 517 nm. The tests were carried out in triplicate. The percentage of free radical scavenging activities was calculated using following Equation (1):

DPPH scavenging activity (%) = (A_0_ − A_1_)/A_0_ × 100% (1)

where A_1_ is the absorbance of the test sample with added EOs and A_0_ is the absorbance value of the absolute ethanol sample without added EOs.

#### 4.4.3. ABTS Free Radical Scavenging Assay

The ABTS solution (7 mmol/L) was prepared according to Cristina et al. [52]. The 2,20-Azino-bis (3-ethylbenzthiazoline-6-sulfonic acid) ABTS working solution was mixed with 50 mL of 2.45 mmol/L K_2_S_2_O_8_ and 50 mL of ABTS. The mixed solution was incubated for 12–16 h at room temperature in the dark, and then diluted with phosphate buffer (PBS, pH 7.0~7.2) to an absorbance of 0.70 ± 0.02 at a wavelength of 405 nm. The Vitamin C and EO solutions at 16, 48, 80, 112 or 144 mg/mL were mixed with 100 µL of ABTS^+^ solution, respectively, and the absorbance was measured at 405 nm. The tests were carried out in triplicate. The percentage of ABTS scavenging effect was calculated using the following Equation (2):

ABTS scavenging activity (%) = (A_0_ − A_1_)/A_0_ × 100% (2)

where A_1_ is the absorbance of the test sample added with EOs and A_0_ is the absorbance value of the absolute ethanol sample without added EOs.

## 5. Statistical Analysis

The IZD data of the EOs were evaluated through analysis of variance (ANOVA). The means were compared by Tukey test (*P* < 0.05) using SAS 9.4 (SAS inst.inc., Cary, Wake, NC, CA, USA). Data for antioxidant activity were analyzed using the SAS 9.4 mixing procedure with model including type of EO, EO concentration and their interactions as fixed effects, three replications of the experiment as random effects. The effect of increasing EO concentration was examined through linear and quadratic orthogonal contrasts using the CONTRAST statement of SAS. Differences were declared significant at *P* ≤ 0.05. The graphs of DPPH radical scavenging rate and ABTS radical scavenging rate were drawn by Origin Pro 2018 software (Northampton, MA, USA).

## 6. Conclusions

The main active compound of the five citrus EOs was d-limonene, but its proportion varied substantially among EOs, ranging from 34.2 to 81.9% of total compounds. The antibacterial activity against pathogenic and beneficial bacteria varied with the type of EO, and the LEO had the best selective antibacterial activity. The LEO and DLEO demonstrated greater antioxidant activity, measured as DPPH and ABTS free radicals compared with other EOs. The high concentration of d-limonene appeared not to be necessarily associated with high antibacterial and antioxidant activity, suggesting that the antibacterial and antioxidant activities might be related to the active component profiles and possibly their synergism effects. Among the five EOs tested, the LEO might have more potential for further consideration for future study.

## Figures and Tables

**Table 1:** Chemical composition profiles of 5 citrus EOs.

No.	RI/Min	Compounds	Composition (% of Total)				
			GEO	SEO	BEO	LEO	DLEO
1	6.917	2-Methyl-5-(1-methylethyl)-bicyclo [3.1.0] hex-2-ene	-	-	0.33	0.62	-
2	7.101	α-Pinene	1.87	2.35	1.63	3.84	2.12
3	7.516	Camphor	-	-	-	0.31	-
4	8.148	1-Isopropyl-4-methylenebicyclo [3.1.0] hexane	3.16	2.45	1.81	-	1.63
5	8.346	β-Pinene	-	-	3.85	13.74	-
6	8.635	β-Laurene	5.93	6.58	1.64	4.20	5.33
7	9.031	Octanal	-	0.40	0.80	-	0.79
8	9.036	3,7-Dimethyl-1,6-octadiene 3-ol carboxylate	-	-	-	1.62	-
9	9.079	β-Rollerene	-	0.28	-	-	1.73
10	9.301	2-Carene	-	-	0.14	-	-
11	9.314	4-Carene	-	-	-	0.87	-
12	10.108	D-Limonene	81.86	78.30	34.23	47.34	80.73
13	10.214	Basilene	-	0.20	0.68	-	-
14	10.252	cis-Beta-Rhodulene	-	-	-	-	0.79
15	10.267	3,7-Dimethyl-1,3,6-octatriene	0.26	-	-	-	-
16	10.519	γ-Pinene	0.17	0.29	4.51	10.55	0.38
17	10.815	P-menthane-3,8-diene	-	-	-	0.58	-
18	11.134	p-Menthane-2,4(8)-diene	-	-	0.30	-	0.46
19	11.159	P-menthane-1,4(8)-diene	-	-	-	1.32	-
20	11.581	Linalool	1.16	2.07	15.18	0.42	0.65
21	11.657	Nonyl aldehyde	0.17	0.28	-	-	-
22	12.077	trans-p-menthane-2,8-dien-1-ol	-	-	-	-	0.87
23	12.267	1,2-Dimethyl-3-(1-methylethenyl) cyclopentanol	-	-	0.30	-	-
24	12.332	cis-4-Isopropenyl-1-methyl-7-oxacyclo [4.1.0] heptane	0.43	-	-	-	-
25	12.444	trans-4-Isopropenyl-1-methyl-7-oxacyclo [4.1.0] heptane	0.33	-	-	-	-
26	12.446	cis-P-menthane-2,8-dien-1-ol					0.36
27	13.235	4-(Acetyloxy)-4-methyl-5-hexenal	-	-	0.37	-	-
28	13.494	2-Isopropenyl-5-methylhexadec-4-enal	-	-	-	-	0.27
29	13.944	α-Pinoresinol	0.10	0.29	0.23	0.41	-
30	13.968	cis-Dihydrocarvone	-	-	-	-	0.18
31	14.176	Decanal	0.12	1.29		0.31	
32	14.301	Octyl acetate	-	-	0.17	-	-
33	14.483	α,4-Dimethyl-3-cyclohexene-1-acetaldehyde	-	-	-	-	0.42
34	14.494	2-Cyclohexen-1-ol, 2-methyl-5-(1-methylethenyl)	0.2	-	-	-	-
35	14.626	3,7-Dimethyl-1,6-octadiene 3-ol formate	-	-	0.69	-	-
36	14.899	5-Isopropenyl-2-methyl-7-oxazolylcyclo [4.1.0] heptan-2-ol	-	-	-	-	0.43
37	14.935	(Z)-3,7-Dimethyl-2,6-octadiene	-	0.22	-	-	-
-38	15.000	cis-Citral				3.18	
39	15.008	Roundup Terpene	-	-	0.15	-	-
40	15.070	Carvone	0.35	0.25	-	-	0.75
41	15.328	Linalyl acetate	2.51	0.21	27.69	-	-
42	15.641	(E)-3,7-Dimethyl-2,6-octadiene	-	0.22	-	-	-
43	15.719	trans-Citral	-	-	-	4.52	-
44	15.768	3,7-Dimethyl-(E)-2,6-octadienal	-	-	0.17	-	-
45	16.074	1-Acetoxymethyl-3-isopropylcyclopentane	-	-	0.66	-	-
46	16.082	Fennel Brain	-	-	-	-	0.35
47	17.461	Pinoresinol α-acetate	-	-	0.27	-	-
48	17.715	2,6-Octadiene -1-ol-3,7-dimethyl acetate	-	-	0.57	-	-
49	17.718	(2Z)-3,7-Dimethyl-2,6-octadienyl acetate	-	-	-	0.99	-
50	18.086	α-Cubene	-	0.33	-	-	-
51	18.15	Geranyl acetate					0.98
52	18.409	2,4-Diisopropyl-1-methyl-cyclohexane	-	-	-	0.13	-
53	18.837	Lauric aldehyde	-	0.24	-	-	-
54	19.050	Sphagnum	0.18	0.19	-	0.48	-
55	19.268	Gamma-elemene	-	-	-	0.67	-
56	19.341	trans-alpha-Bergamotine	-	-	0.78	-	-
57	19.768	trans-β-Farnesene	-	-	0.18	-	-
58	19.825	Humulene	-	-	-	0.45	-
59	20.41	cis-β-Farnesene	-	-	0.11	-	-
60	20.414	4,11,11-Trimethyl-8-methylenebicyclo [7.2.0 ndecylene-4-ene	-	-	-	0.15	-
61	20.592	1,2,3,5,6,7,8,8a-Octahydro-1,8a-dimethyl-7-(1-methylethenyl) naphthalene	-	0.44	-	0.21	-
62	20.840	α-Farnesene	-	-	-	1.42	-
63	20.915	β-Bisabolene	-	-	0.55	-	-
64	21.106	1-Isopropyl-4,7-dimethyl-1,2,3,5,6,8a-hexahydronaphthalene	-	0.38	-	-	-
65	23.779	Triethyl citrate	-	-	-	0.56	-
66	24.517	2,6,11-Dodecanetraldehyde	-	0.22	-	-	-
67	26.512	Round pomelo ketone	0.33	-	-	-	-
68	28.548	P-menthol-1(7),8-diene	-	-	-	0.33	-
69	28.944	Mesocamphorine	-	-	-	0.34	-
70	29.417	5,7-Dimethoxy-2H-1-benzopyran-2-one	-	-	0.21	-	-
71	29.523	For camphorine	-	-	-	0.20	-
72	30.469	4-Methoxy-7H-furo [3,2-g][1] benzopyran-7-one	-	-	0.24	-	-
73	31.373	(S, E)-2,5-Dimethyl-4-vinylhexa-2,5-dien-1-yl acetate	-	-	-	0.24	-
74	33.268	(S)-8-((3,3-Dimethyloxiran-2-yl) methyl)-7-methoxy-2H-chromen-2-one	0.18	-	-	-	-
75	38.517	2,7-Dimethyl-2,7-diol	0.69	-	-	-	-
76	43.278	Tris(2,4-di-tert-butylphenyl) phosphate	-	-	0.28	-	0.78
77	44.631	4′,5,6,7,8-Pentamethoxyflavone	-	0.88	-	-	-
78	46.399	3,5,6,7,8,3′,4′-Heptyloxyflavone	-	0.79	-	-	-
79	46.632	3′,4′,5,6,7,8-Hexamethoxyflavone	-	0.85	-	-	-
		Total	100	100	100	100	100
		monoterpene olefins	90.27	89.18	48.3	86.58	96.19

Note: “-” indicates that EO does not contain this component. GEO: grapefruit essential oil, SEO: sweet orange essential oil, BEO: bergamot essential oil, LEO: lemon essential oil, DLEO: d-limonene essential oil.

**Table 2:** MIC and MBC of citrus EOs against different bacteria.

	Bacterial Strains	EOs (mg/mL)				
		GEO	SEO	BEO	LEO	DLEO
MIC	*E. coli*	2.5	20	20	5	10
	*Salmonella*	5	2.5	5	10	1.25
	*L. acidophilus*	80	80	80	80	40
MBC	*E. coli*	40	80	80	10	40
	*Salmonella*	160	80	80	40	40
	*L. acidophilus*	160	160	160	160	80

Experiments were carried out in triplicate. GEO: grapefruit essential oil, SEO: sweet orange essential oil, BEO: bergamot essential oil, LEO: lemon essential oil, DLEO: d-limonene essential oil.

**Table 3:** Inhibition zone diameter (IZD, mm) of citrus EOs.

Bacterial Strains	EOs (mg/mL)					SEM	*p* Value
	GEO	SEO	BEO	LEO	DLEO		
*E. coli*	10.67	11.33	12.67	14.33	12.33	0.84	0.09
*Salmonella*	12.33	11.33	11.33	12.33	11.00	0.60	0.41
*L. acidophilus*	14.33 ^a^	13.33 ^ab^	10.33 ^c^	8.67 ^d^	11.67 ^bc^	0.56	<0.001

Zone of growth inhibition values are expressed as the mean of at least three experiments in mm, including a disc diameter of 6.0 mm. According to ANOVA and Turkey test, different superscript letters in the same column showed differences (*P* < 0.05). GEO: grapefruit essential oil, SEO: sweet orange essential oil, BEO: bergamot essential oil, LEO: lemon essential oil, DLEO: d-limonene essential oil. SEM: standard error of measurement.

## Data Availability

Data are contained within the article.

## Sample Availability

Samples of the compounds are available from the authors.
